# Sacituzumab-induced severe or febrile neutropenia and G-CSF utilization and cost for advanced HER2-negative breast cancer: a single-center retrospective analysis

**DOI:** 10.1007/s10549-026-07971-z

**Published:** 2026-04-17

**Authors:** Grace Tam, Donald Waddell, Jincong Q. Freeman, Nan Chen, Heng Yang

**Affiliations:** 1 Department of Pharmacy, University of Chicago Medicine, 5841 S Maryland Ave, Chicago, IL 60637 USA; 2 Department of Public Health Sciences, University of Chicago, Chicago, IL USA; 3 Cancer Prevention and Control Research Program, University of Chicago Comprehensive Cancer Center, Chicago, IL USA; 4 Center for Health and the Social Sciences, University of Chicago, Chicago, IL USA; 5 Section of Hematology & Oncology, Department of Medicine, University of Chicago, Chicago, IL USA

**Keywords:** Sacituzumab, Severe neutropenia, Febrile neutropenia, G-CSF utilization, G-CSF cost, HER2-Negative advanced breast cancer

## Abstract

**Purpose:**

Severe neutropenia (SN) and febrile neutropenia (FN) are clinically significant safety concerns of sacituzumab govitecan-hziy (SG). We sought to compare the rates of SN and FN from clinical trials to real-world experience, correlate factors with SN and FN, and quantify granulocyte colony-stimulating factor (G-CSF) use in HER2-negative (HER2-) metastatic breast cancer (mBC).

**Methods:**

We performed a retrospective analysis of patients treated with SG for advanced HER2- BC at a single US institution with a diverse patient population. The rates of SN and FN, stratified by receptor status, were compared to their respective phase III clinical trials. Multivariable logistic regression was used to evaluate factors associated with SN and FN.

**Results:**

Of 87 patients treated with SG, 10 patients received primary prophylaxis. Of the 77 patients who didn’t receive primary G-CSF prophylaxis, 49% and 3.9% patients developed SN and FN, respectively. SN and FN rates did not differ in the HR + /HER- mBC or mTNBC subgroups compared to clinical trials. Factors evaluated in this study were not shown to be associated with SN. Overall, 44% (38/87) required a dose interruption or reduction due to SN or FN. 11% (10/87) and 60% (52/87) received G-CSF as primary and secondary prophylaxis respectively, with a total G-CSF drug cost of $2.1 million.

**Conclusions:**

SG-induced SN and FN rates were similar to those reported in clinical trials, both in the HR + /HER- mBC and mTNBC groups. Due to small sample size, we did not identify any statistically significant risk factors of SG-associated SN. Our study provided insights into G-CSF use patterns and costs in real-world SG therapy.

## Introduction

Sacituzumab govitecan-hziy (SG) is a trophoblastic cell surface antigen-2 (TROP-2)–directed antibody–drug conjugate. It has been used to treat metastatic triple-negative breast cancer (mTNBC) and hormone receptor-positive/HER2-negative (HR +/HER2-) metastatic breast cancer (mBC) in the second or later line setting [1]. In the ASCENT trial, SG significantly improved progression-free survival (PFS) and overall survival (OS) compared to single-agent chemotherapy (eribulin, vinorelbine, capecitabine, or gemcitabine) in refractory mTNBC patients, with a median PFS of 5.6 months vs 1.7 months, and a median OS of 12.1 months vs 6.7 months [2]. In HR +/HER2- mBC, the phase III TROPiCS-02 trial similarly showed improved median PFS (5.5 months vs 4.0 months) and OS (14.1 months vs 11.2 months) with SG compared to chemotherapy in patients with endocrine-resistant, heavily pretreated disease. [3]

The most common adverse effects of SG include neutropenia and gastrointestinal symptoms, such as nausea, vomiting, and diarrhea. In ASCENT and TROPiCS-02 trials, the reported incidence of febrile neutropenia (FN) and severe (grade 3 and 4) neutropenia (SN) were 4–6% and 51%, respectively. [2, 3] Due to the risk of severe, life-threatening, or fatal neutropenia, the US prescribing information of SG includes a black box warning for this side effect [1]. Secondary prophylaxis with granulocyte colony-stimulating factor (G-CSF) is recommended to mitigate the risk of SN and FN [4, 5]. Primary G-CSF prophylaxis may be considered for patients with risk factors [4, 5]. According to the National Comprehensive Cancer Network (NCCN) Hematopoietic Growth Factors guideline, these risk factors include prior chemotherapy or radiation, persistent neutropenia, bone marrow involvement by tumor, poor performance status, recent surgery, liver or renal dysfunction, and/or ages > 65 receiving full-dose chemotherapy [4]. In addition, patients with the UGT1A1 *28/*28 genotype have been shown to experience higher rates of SG-related SN and FN than those with heterozygous (*1/*28) and wild type (*1/*1) genotypes, indicating the impact of UGT1A1 status on the risk of SG-induced neutropenia, although this testing is not routinely performed at our center and others. [6]

It is important to note that the large clinical trials of SG excluded patients with poor functional status, multiple comorbidities, or those who were not trial candidates due to limited psychosocial support. In fact, these individuals may be at a higher risk for SG-induced toxicities in the real-world setting. Reflecting this concern, the NCCN reclassified SG from low to intermediate FN risk on October 11th 2024, acknowledging a potential real-world FN incidence of 10–20%, which exceeds the rates reported in clinical trials [4]. Therefore, this study aims to evaluate the safety profile of SG in real-world practice, identify patient-specific risk factors for SG-related SN and FN, evaluate the clinical consequences of these toxicities, and assess the associated G-CSF costs of SG therapy in advanced HER2- breast cancer.

## Methods

### Study design and population

This was a single-institution, retrospective study. Data for this analysis were collected from adult patients aged ≥ 18 years who received at least 1 dose of SG for mTNBC or HR +/HER2- mBC at the University of Chicago Medicine (UCM) health system between April 1 st, 2020 and September 1 st, 2024. Electronic health record reports were generated to identify eligible patients. All patients were female. Exclusion criteria included patients receiving SG for an indication other than breast cancer and concomitant treatment with biological agents and/or immunotherapy. This retrospective study was approved by the UCM’s institutional review board and is in compliance with the Declaration of Helsinki and International Conference on Harmonization Guidelines for Good Clinical Practice. Patient informed consent was not needed due to the retrospective nature of this study.

### Measures and definitions

The primary objective of this single-center retrospective study was to evaluate SN (defined as grade 3 and 4 neutropenia) and FN (defined as grade 3 or 4 neutropenia with single temperature of > 38.3 degrees C/101 degrees F or sustained temperature of > 38 degrees C/100.4 degrees F) in patients treated with SG for mTNBC or HR +/HER2- mBC. Neutropenia and FN were graded using Common Terminology Criteria for Adverse Events (CTCAE) Version 5.0. Demographic and clinical factors also included age group (≤ 65 or > 65 years), self-reported race (non-Hispanic Black [NHB], non-Hispanic White [NHW], or Other), insurance type (Medicaid, Medicare or private insurance), the Eastern Cooperative Oncology Group (ECOG) performance status (0–1 or > 1), number of prior lines of myelosuppressive chemotherapy agents (< 3 or ≥ 3), baseline absolute neutrophil count (ANC; ≤ 50% or > 50% quartile), liver function (total bilirubin level < 2 or ≥ 2), renal function (creatinine clearance < 50 or ≥ 50), metastasis to bone, recent surgery within 3 months, recent radiation to bone, and SG starting dose (full or reduced dose). Secondary objectives included examining the SN or FN associated treatment consequences such as SG dose interruption or reduction, patient hospitalization, and G-CSF utilization. Dose interruption was defined as delay of seven days of more from planned treatment. Pegfilgrastim and filgrastim were the only G-CSF products used at UCM. The G-CSF utilization analysis included both brand-name and biosimilar versions of these agents, regardless of whether they were administered by patients themselves or by the facility. The cost of G-CSF was estimated using the wholesale acquisition cost of each specific product. G-CSF administration associated cost was not included in this study. Hospitalization costs were calculated by multiplying the number of days hospitalized by an estimated cost of $10,000 per day, based on historical chemotherapy induced FN in breast cancer cost data, adjusted to 2022 U.S. dollars using the Consumer Price Index (CPI) Hospital Services. [7–9]

### Statistical analysis

To describe the study sample, we computed descriptive statistics and evaluated the distributions of the demographic and clinical characteristics, overall and by SN. Group comparisons were conducted using student’s *t*-test, Wilcoxon rank-sum, Pearson’s chi-squared, or Fisher’s exact tests whichever was appropriate. Similar statistics were computed and used to compare the rates of SN and FN between our real-world patient sample to that of clinical trials. To examine factors associated with SN (vs no SN), we fit univariate and multivariable logistic regression models. The multivariable-adjusted regression model included age group, race, receptor status, ECOG performance status, baseline ANC, prior lines of myelosuppressive chemotherapy, bone metastasis, surgery status, hepatic function, and starting dose. Adjusted odds ratios (AOR) and 95% confidence intervals (95% CI) were calculated and reported. A 2-sided *p*-value of < 0.05 was considered statistically significant. All data analyses were conducted using Stata version 18.0 (StataCorp LLC, College Station, TX).

## Results

### Patient characteristics

A total of 87 eligible patients treated with SG for mTNBC or HR +/HER2- mBC were included in this study. 10 patients received primary G-CSF prophylaxis. The baseline characteristics of the 77 patients who did not receive primary prophylaxis are presented in Table 1, highlighting differences between those with and without SN. Among the 77 patients, 52 (68%) had neutropenia and 38 (49%) had SN. The median age was 56.4 (IQR, 47.3–65.4) years. Twenty-eight (36%) patients were NHB, 38 (49%) were NHW, and 11 (14%) of other races. There were 59 (77%) and 18 (23%) patients with mTNBC and HR +/HER2- mBC, respectively. The ECOG performance status was 0 to 1 in 68% of the patients. 40% had ≥ 3 lines of prior myelosuppressive chemotherapy.

**Table 1:** Demographic and clinical characteristics of patients with advanced HER2- breast cancer who received SG without primary G-CSF prophylaxis

Variable	All patients	Patients without severe neutropenia, N (%) (No neutropenia, grade 1 and 2)	Patients with severe neutropenia, N (%) (Grade 3 and 4 neutropenia)	
	*N* = 77	*N* = 39 (50.6)	*N* = 38 (49.4)	*p*-value ^a^
Age in years				
Mean (SD)	56.4 (13.0)	55.5 (13.2)	57.4 (12.9)	0.52
Median (IQR)	56.4 (47.3, 65.4)	56.4 (43.4, 64.4)	57.1 (49.4, 67.3)	0.56
Age group				
≤ 65 years	57 (74)	30 (77)	27 (71)	0.61
> 65 years	20 (26)	9 (23)	11 (29)	
Race				
Non-Hispanic Black (NHB)	28 (36)	11 (28)	17 (45)	0.01
Non-Hispanic White (NHW)	38 (49)	18 (46)	20 (53)	
Other	11 (14)	10 (26)	1 (3)	
Insurance type				
Medicare or private	64 (83)	31 (79)	33 (87)	0.54
Medicaid	13 (17)	8 (21)	5 (13)	
Receptor status				
HR +/HER2- mBC	18 (23)	7 (18)	11 (29)	0.29
mTNBC	59 (77)	32 (82)	27 (71)	
ECOG				
> 1	25 (32)	15 (38)	10 (26)	0.33
0 or 1	52 (68)	24 (62)	28 (74)	
Baseline ANC (mm^3^)*				
> 4000	37 (50)	17 (46)	20 (54)	0.64
≤ 4000	37 (50)	20 (54)	17 (46)	
Prior lines of myelosuppressive chemotherapy ^b^				
< 3	46 (60)	23 (59)	23 (61)	1.00
≥ 3	31 (40)	16 (41)	15 (39)	
Bone metastasis				
No	41 (53)	22 (56)	19 (50)	0.65
Yes	36 (47)	17 (44)	19 (50)	
Prior radiation to the bone				
No	76 (99)	38 (97)	38 (100)	1.00
Yes	1 (1)	1 (3)	0 (0)	
Recent surgery within 3 months				
No	73 (95)	37 (95)	36 (95)	1.00
Yes	4 (5)	2 (5)	2 (5)	
Renal function (mL/min)				
Creatinine clearance ≥ 50	76 (99)	39 (100)	37 (97)	0.49
Creatinine clearance < 50	1 (1)	0 (0)	1 (3)	
Hepatic function (mg/dL)				
Total bilirubin level < 2	73 (95)	37 (95)	36 (95)	1.00
Total bilirubin level ≥ 2	4 (5)	2 (5)	2 (5)	
SG starting dose				
Reduced dose	9 (12)	5 (13)	4 (11)	1.00
Full dose	68 (88)	34 (87)	34 (89)	

*SG* sacituzumab govitecan, *SD* standard deviation, *IQR* interquartile range, *HR* hormone receptor, *HER2* human epidermal growth factor receptor 2, *mBC* metastatic breast cancer, *mTNBC* metastatic triple-negative breast cancer, *ECOG* the Eastern Cooperative Oncology Group, *ANC* absolute neutrophil counts, *G-CSF* granulocyte colony-stimulating factor

^*^Three patients with unknow baseline ANC due to transfer of care

^a^ *P*-value was calculated using *t*-test, Wilcoxon rank-sum, or Fisher’s exact tests, as appropriate

^b^ Prior lines of myelosuppressive chemotherapy includes PARP inhibitor within 12 months if curative setting

### Rates of SN and FN

The rates of SN and FN in both HR +/HER2- mBC and mTNBC treated with SG were similar between our real-world study and clinical trials (Table 2 and Table 3). Overall, 49% of SN and 3.9% of FN were observed in this study. For HR +/HER2- mBC patients, we found 50% of the patients had SN compared to 51% in the TROPiCS-02 trial [2]. In HR +/HER2-mBC patients, we had no FN cases recorded compared to a 5% rate in the TROPiCS-02 trial [2]. Likewise, in mTNBC patients, we saw similar rates of SN, 49% in our study compared to 51% in the ASCENT trial [3]. Our rates of FN in mTNBC also did not differ from the ASCENT trial (5% vs 6%) [3]. Prolonged neutropenia (neutropenia lasting > 7 days) were recorded in 9 patients. There were two cases of FN that required hospitalization resulting in an estimated total cost of $280,000. No SN or FN related death were reported in this study.

**Table 2:** Neutropenia rate in patients with HER2- mBC who received SG without primary G-CSF prophylaxis: comparisons between studies

		Patients without severe neutropenia, *N* (%) (No neutropenia, grade 1 and 2 neutropenia)	Patients with severe neutropenia, *N* (%) (Grade 3 and 4 neutropenia)	*p*-value ^a^
In patients with metastatic TNBC				
	Our study (*N* = 59)	30 (51)	29 (49)	0.78
	ASCENT trial (*N* = 258)	126 (49)	132 (51)	
In patients with HR +/HER2- metastatic breast cancer				
	Our study (*N* = 18)	9 (50)	9 (50)	1.00
	TROPiCS-02 trial (*N* = 268)	132 (49)	136 (51)	

*SG* sacituzumab govitecan, *TNBC* triple-negative breast cancer, *HR* hormone receptor, *HER2* human epidermal growth factor receptor 2, *G-CSF* granulocyte colony-stimulating factor

^a^ *P*-value was calculated using the Pearson’s Chi-squared test

**Table 3:** Rate of febrile neutropenia in patients with advanced HER2- breast cancer who received SG without primary G-CSF prophylaxis: comparison between studies

		Patients without febrile neutropenia, *N* (%)	Patients with febrile neutropenia, *N* (%)	*p*-value ^a^
In patients with metastatic TNBC				
	Our study (*n* = 59)	56 (95)	3 (5)	1.00
	ASCENT trial (*n* = 258)	243 (95)	15 (6)	
In patients with HR +/HER2- metastatic breast cancer				
	Our study (*n* = 18)	18 (100)	0	1.00
	TROPiCS-02 trial (*n* = 268)	254 (95)	14 (5)	

*mBC* metastatic breast cancer, *SG* sacituzumab govitecan, *TNBC* triple-negative breast cancer, *HR* hormone receptor, *HER2* human epidermal growth factor receptor 2, *G-CSF* granulocyte colony-stimulating factor

^a^ *P*-value was calculated using the Fisher’s exact test

### Associated factors with SN

NHB patients had a relatively higher rate of SN than NHW (61% vs 53%) (Table 4); however, this difference was not a statistically significant (AOR = 1.58, 95% CI = 0.51–4.90). Patients who identified as other races had lower odds of SN than NHW (AOR = 0.08, 95% CI = 0.01–0.78), which warrants further investigation. Other factors, including age, receptor status, ECOG performance status, baseline ANC, prior lines of myelosuppressive chemotherapy, bone metastasis, recent surgery (within 3 months), liver and renal function, and SG starting dose, were also not shown to be significantly associated with SN in this study.

**Table 4:** Associated factors with severe neutropenia among patients with advanced HER2- breast cancer who received SG

		Logistic regression			
Variable	Patients with severe neutropenia, N (%) (grade 3 and 4 neutropenia)	Unadjusted OR (95% CI)	*p*-value	Adjusted OR (95% CI) ^a^	*p*-value
Age group					
≤ 65 years	27 (47)	1.0 (reference)		1.0 (reference)	
> 65 years	11 (55)	1.36 (0.49–3.78)	0.56	1.11 (0.32–3.89)	0.87
Race					
Non-hispanic black (NHB)	17 (61)	1.39 (0.52–3.74)	0.51	1.58 (0.51–4.90)	0.43
Non-hispanic white (NHW)	20 (53)	1.0 (reference)		1.0 (reference)	
Other	1 (9)	0.09 (0.01–0.77)	0.03	0.08 (0.01–0.78)	0.03
Insurance type					
Medicare or private	33 (52)	1.0 (reference)		NA	NA
Medicaid	5 (38)	0.59 (0.17–1.99)	0.39	NA	NA
Receptor status					
HR +/HER2- mBC	11 (61)	1.86 (0.63–5.47)	0.26	2.13 (0.53–8.52)	0.29
mTNBC	27 (46)	1.0 (reference)		1.0 (reference)	
ECOG					
> 1	10 (40)	1.0 (reference)		1.0 (reference)	
0 or 1	28 (54)	1.75 (0.66–4.61)	0.26	1.57 (0.44–5.66)	0.49
Baseline ANC*					
> 50% quartile	20 (54)	1.38 (0.55–3.45)	0.49	1.11 (0.39–3.16)	0.85
≤ 50% quartile	17 (46)	1.0 (reference)		1.0 (reference)	
Prior lines of myelosuppressive chemotherapy ^b^					
< 3	23 (50)	1.0 (reference)		1.0 (reference)	
≥ 3	15 (48)	0.94 (0.39–2.33)	0.89	1.27 (0.41–4.00)	0.68
Bone metastasis					
No	19 (46)	1.0 (reference)		1.0 (reference)	
Yes	19 (53)	1.29 (0.53–3.17)	0.57	0.97 (0.31–3.04)	0.96
Prior radiation to the bone					
No	38 (50)	NA	NA	NA	NA
Yes	0 (0)	NA	NA	NA	NA
Recent surgery within 3 months					
No	36 (49)	1.0 (reference)		1.0 (reference)	
Yes	2 (50)	1.03 (0.14–7.69)	0.98	0.85 (0.07–9.94)	0.90
Renal function (mL/min)					
Creatinine clearance ≥ 50	37 (49)	NA	NA	NA	NA
Creatinine clearance < 50	1 (100)	NA	NA	NA	NA
Hepatic function (mg/dL)					
Total bilirubin level < 2	36 (49)	1.0 (reference)		1.0 (reference)	
Total bilirubin level ≥ 2	2 (50)	1.03 (0.14–7.69)	0.98	0.93 (0.11–7.65)	0.95
SG starting dose					
Reduced dose	4 (44)	1.0 (reference)		1.0 (reference)	
Full dose	34 (50)	1.25 (0.31–5.06)	0.75	2.07 (0.44–9.63)	0.35

*SG* sacituzumab govitecan, *No.* number, *NA* not applicable, *HR* hormone receptor, *HER2* human epidermal growth factor receptor 2, *mBC* metastatic breast cancer, *mTNBC* metastatic triple-negative breast cancer, *ECOG* the Eastern Cooperative Oncology Group, *ANC* absolute neutrophil counts, *OR* odds ratio, *CI* confidence interval

^*^One patient with unknown baseline ANC due to transfer of care

^a^ Adjusted for all variables in the table, except for insurance type, prior radiation to the bone, and renal function

### SG dose reductions and interruptions

A total of 1,152 SG doses were recorded over 614 cycles. Among the 87 patients, the median number of SG doses administered was 10 (range, 1–90) over 5 cycles (range,1–45). 90% (78/87) of patients started on a full dose of SG at 10 mg/kg on days 1 and 8 of a 21-day cycle. Those starting at reduced doses (with 25% or 50% initial dose reduction) were due to frailty, anemia, liver dysfunction, or a history of neutropenia. Overall, 26% (23/87) required dose reduction and 38% (33/87) required a dose interruption due to SN or FN (Table 5). 44% (38/87) required either a dose reduction or interruption due to SN or FN. Some of these patients were limited by insurance coverage of G-CSF products, leading to greater dose reductions or interruptions.

**Table 5:** Sacituzumab govitecan dose reduction and interruption related to severe neutropenia or febrile neutropenia

	All patients received SG treatment (*N* = 87)	Patient received SG treatment without primary G-CSF prophylaxis (*N* = 77)
	N (%)	N (%)
Dose reduction due to SN or FN	23 (26)	21 (27)
Dose interruption due to SN or FN	33 (38)	32 (42)
Dose reduction or dose interruption due to SN or FN	38 (44)	36 (47)
Prolonged neutropenia (> 7 days)	9 (10)	9 (12)

*SG* sacituzumab govitecan, *SN* severe neutropenia, *FN* febrile neutropenia, *G-CSF* granulocyte colony-stimulating factor

Beyond SN and FN, other factors also led to dose reductions or interruptions. Dose reductions for any cause occurred in 52% of patients (45/87), with 7% (6/87) requiring a second dose reduction. Reasons for dose reductions other than SN and FN included thrombocytopenia or anemia in two patients and grade 3 or 4 diarrhea in nine patients. The remaining cases were due to fatigue, nausea, elevated liver function tests, weakness, neuropathy, abdominal pain or rash. A similar pattern was observed with dose interruptions: 63% (55/87) patients experienced interruptions due to the same toxicities noted above. Due to our limited sample size, we were unable to assess associations between SN and other SG-induced toxicities.

### G-CSF utilization

In our study, 10 out of the 87 (11%) patients who received SG started treatment with primary G-CSF prophylaxis before the initial 2024 updates to the NCCN Hematopoietic Growth Factors Guideline that reclassified sacituzumab’s FN risk to intermediate. However, all 10 patients had at least two risk factors, consistent with NCCN’s recommendation to consider G-CSF for patients receiving low-risk regimens. The most common risk factor among those 10 patients was prior chemotherapy requiring G-CSF support, followed by bone marrow involvement by tumor, persistent neutropenia, prior radiation, and ages > 65 receiving full chemotherapy dose intensity.

Among the remaining 77 patients, 52 (68%) received secondary prophylaxis. This included G-CSF use for 9 of 14 (64%) patients with grade 1 or 2 neutropenia. An additional 8 patients had an unknown grade of neutropenia at G-CSF initiation due to transfer of care. The remaining 35 patients utilized G-CSF for SN. Three patients with SN did not receive G-CSF. Among patients who received primary prophylaxis, 2 (20%) had Medicaid, 3 (30%) had Medicare, and 5 (50%) were privately insured. In comparison, the overall study population included 41 (47%) patients with private insurance, followed by 27 (31%) with Medicare and 15 (17%) patients with Medicaid. There was no apparent association between insurance type and the use of primary or secondary G-CSF prophylaxis in this study.

For the 10 patients who received primary G-CSF prophylaxis, all patients were on pegfilgrastim starting cycle 1, to be administered after day 8 of SG. One patient added filgrastim after day 1 of SG for subsequent cycles. Of the 52 patients who received secondary G-CSF prophylaxis, 37 (71%) started during cycle 1 after receiving the first dose of SG, 10 (19%) started during cycle 2, 3 (5.8%) started during cycle 3 or later while 2 patients had an unknown start date due to a lack of clinical documentation when they transferred from other institutions. Regarding G-CSF type, 15 (29%) received filgrastim only, 26 (50%) received pegfilgrastim only, and 11 (21%) received both filgrastim and pegfilgrastim. The total drug cost of G-CSF use in this study was approximately $2.1 million. Averaged over 614 cycles of SG, 1.9 doses of G-CSF were used per cycle, at a cost of $3,344.

## Discussion

Neutropenia is one of the major safety concerns of SG, which may result in therapy interruption, dose reduction, hospitalization and potentially life-threatening complications. This retrospective study is the first to evaluate risk factors of SN and FN, G-CSF use patterns, and costs associated with SG-induced neutropenia among patients with HR +/HER2- mBC or mTNBC seen at UCM. We aimed to assess how SG treatment was tolerated in real-world practice. Overall, our study did not show significant differences in the rates of SN and FN compared to those reported in the landmark, phase III studies. Our cohort was racially diverse, with 36% of patients identifying as NHB compared to 12% in ASCENT study and 3% in TROPiCS-02 trial [2, 3]. Currently, data on whether there are racial differences in SG-induced neutropenia among breast cancer patients are inconsistent [10, 11]. This study may shed some light on race and SG-associated SN and FN. Compared to the landmark trials of SG which only included patients with ECOG of 0 or 1 [2, 3], our study included 25 (32%) of patients with an ECOG ≥ 2. Poor performance status is known to increase the risk of FN in patients receiving chemotherapy [12–15]. Our study did not show higher rates of SN and FN in patients with poor performance status, possibly due to the smaller sample size. It may also reflect real-world practice, where some patients with poor functional status received upfront SG dose reductions, which are not permitted in clinical trials.

The incidence of SN and FN may increase in patients with certain pre-existing risk factors. Besides race and functional status, this study also evaluated other risk factors outlined in the NCCN guideline. NHB race, HR +/HER- receptor status, prior use of three or more lines of myelosuppressive chemotherapy, and full starting dose showed numerical trends in leading to higher odds of SN or FN. However, we couldn’t identify factors showing statistically significant associations in predicting SN and FN. This is most likely due to the small sample size of this single center study. These findings may help guide future, larger studies in identifying patients at increased risk of SG-induced SN or FN who may benefit from primary G-CSF prophylaxis. A previous study has shown that patients with reduced UGT1A1 activity are at increased risk for SN and FN [6]. UGT1A1 activity was not evaluated in the subgroup analysis since UGT1A1 status was not routinely obtained in real-world practice for patients not on clinical trials.

Prevention and early management of SN and FN are essential to avoid related complications. According to SG prescribing information and the NCCN guideline, primary or secondary prophylaxis with G-CSF is recommended during SG treatment as G-CSF use is known to reduce the incidence, duration, and severity of FN [4]. The high use of G-CSF likely contributed to the relatively low FN rate observed in landmark clinical trials and in this real-world study, despite the high risk of SN associated with SG. In our study, most (71.6%) patients were given G-CSF support. In prior phase III trials, 49–54% of SG-treated patients received G-CSF. [2, 3] However, in clinical practice, obtaining primary G-CSF prophylaxis is often challenging due to insurance restrictions, even for patients with preexisting risk factors for FN. Another challenge is the difficulty of establishing a standardized G-CSF plan. A combination of short-acting G-CSF after Day 1 and long-acting G-CSF after Day 8 may be needed given SG’s Day 1 and Day 8 dosing schedule in a 21-day cycle, however insurance may not approve the concurrent use of two different G-CSF products. It is also inconvenient to use this combination or use short-acting G-CSF after both Day 1 and Day 8 due to multiple self-injections.

Additionally, the cost associated with G-CSF therapy may be significant and may prevent patients from obtaining treatment. These practical challenges prompted us to conduct this study to evaluate G-CSF utilization patterns and the associated costs in patients with SG-induced neutropenia. In our study, secondary G-CSF prophylaxis was used more frequently than primary G-CSF prophylaxis, 60% (52/87) vs 11.5% (10/87), although no apparent association was observed between insurance type and the use of primary or secondary G-CSF. In terms of G-CSF type, use of filgrastim alone, pegfilgrastim alone, or a combination of both was observed. The choice of G-CSF was most likely influenced by patient and physician preferences, insurance coverage, and cost considerations. The average drug cost of G-CSF observed in this study was $3,344 per SG treatment cycle, which could represent a significant financial burden for both patients and the healthcare system. This cost analysis included only the cost of G-CSF. G-CSF administration costs were not included and could vary depending on patients’ insurance coverage.

This study has several limitations. This was a single-center study with a limited sample size. The retrospective design could only allow us to collect data and information available in progress notes and laboratory tests. Inclusion of patients who transferred into or out of our clinic further reduced completeness of care details due to restricted access to outside records. In addition, the severity of neutropenia could not be determined in patients who initiated G-CSF before transfer, likely leading to underestimation of the true incidence of SN. Nonetheless, our findings on SN or FN risk factors, SG treatment modifications, and G-CSF use contribute to a better understanding of the real-world application of SG.

## Conclusions

In this single center retrospective study, SG-induced SN and FN rates were similar to those reported in clinical trials, both in the HR +/HER- mBC and mTNBC groups. Due to limited sample size, we did not identify any statistically significant risk factors of SG-associated SN. Future research is needed to better define the risk factors for SG-induced SN and FN. In this real-world study, SG-induced neutropenia was one of the leading causes of SG dose reduction and treatment interruption. G-CSF prophylaxis was widely used in preventing SG-induced SN and FN. Given the substantial financial burden of G-CSF use associated with SG treatment, further efforts are needed to explore more cost-effective strategies for G-CSF use in this setting.

## Data Availability

Data for our analysis cannot be shared publicly due to the confidentiality and privacy of patient information. However, the data can be de-identified and requested from the corresponding authors with a reasonable request.
